# SciKit digital health package for accelerometry-measured physical activity: comparisons to existing solutions and investigations of age effects in healthy adults

**DOI:** 10.3389/fdgth.2023.1321086

**Published:** 2023-11-27

**Authors:** Wenyi Lin, F. Isik Karahanoglu, Charmaine Demanuele, Sheraz Khan, Xuemei Cai, Mar Santamaria, Junrui Di, Lukas Adamowicz

**Affiliations:** ^1^ AI/ML Quantitative and Digital Sciences, Global Biometrics and Data Management, Pfizer Research and Development, Pfizer Inc, Cambridge, MA, United States; ^2^ Digital Sciences & Translational Imaging, Early Clinical Development, Pfizer Research and Development, Pfizer Inc, Cambridge, MA, United States

**Keywords:** physical activity, accelerometer, continuous monitoring, remote monitoring, age effects

## Abstract

**Introduction:**

Accelerometry has become increasingly prevalent to monitor physical activity due to its low participant burden, quantitative metrics, and ease of deployment. Physical activity metrics are ideal for extracting intuitive, continuous measures of participants’ health from multiple days or weeks of high frequency data due to their fairly straightforward computation. Previously, we released an open-source digital health python processing package, SciKit Digital Health (SKDH), with the goal of providing a unifying device-agnostic framework for multiple digital health algorithms, such as activity, gait, and sleep.

**Methods:**

In this paper, we present the open-source SKDH implementation for the derivation of activity endpoints from accelerometer data. In this implementation, we include some non-typical features that have shown promise in providing additional context to activity patterns, and provide comparisons to existing algorithms, namely GGIR and the GENEActiv macros. Following this reference comparison, we investigate the association between age and derived physical activity metrics in a healthy adult cohort collected in the Pfizer Innovation Research Lab, comprising 7–14 days of at-home data collected from younger (18–40 years) and older (65–85 years) healthy volunteers.

**Results:**

Results showed that activity metrics derived with SKDH had moderate to excellent ICC values (0.550 to 1.0 compared to GGIR, 0.469 to 0.697 compared to the GENEActiv macros), with high correlations for almost all compared metrics (>0.733 except vs GGIR sedentary time, 0.547). Several features show age-group differences, with Cohen’s d effect sizes >1.0 and p-values < 0.001. These features included non-threshold methods such as intensity gradient, and activity fragmentation features such as between-states transition probabilities.

**Discussion:**

These results demonstrate the validity of the implemented SKDH physical activity algorithm, and the potential of the implemented PA metrics in assessing activity changes in free-living conditions.

## 1. Introduction

Inertial measurement units (IMUs) have gained popularity and became one of the best solutions for continuous monitoring of populations in free-living environments (1). This has led to various algorithms being developed to extract digital metrics that quantify physical activity (PA) from continuous accelerometer recordings. The simplest of these methods compute general summary metrics of physical activity throughout the day, oftentimes through energy-expenditure based thresholds applied to lightly filtered and transformed data (2). These physical activity metrics provide a high-level summary of how active a user is day-to-day, without requiring more sophisticated processing to estimate metrics from gait, for example. Additionally, the typical wrist-based placement to obtain these measures of physical activity provides an easy, convenient, and relatively comfortable mounting spot for the IMUs which can take the place of or be integrated with watches or other wrist bands (3).

The simplicity and convenience have therefore led to previous work into physical activity metrics, from early work on circadian rhythm (4, 5), to more recent work investigating physical activity trends in large populations (6, 7). In practice, most algorithms that estimate these physical activity metrics are similar; first they compute a summary measure of acceleration magnitude (8, 9), often with light signal filtering, then threshold into multiple different bins (sedentary, light, moderate, and vigorous physical activity) based on metabolic equivalent tasks (METs), and compute the amount of time in each of the bins (10–15). While easy to understand and interpret, these four activity bin thresholds are empirically based, resulting in discrepancies in threshold values, even among the same populations, as well as largely different thresholds for healthy children compared to healthy adults (13, 14). These population threshold differences pose problems when working with non-healthy populations, as the thresholds may not allow for fully sensitive detection of changes in activity level. However, there have been suggested ways to move away from threshold-based digital metrics, such as examining the decreasing amount of time spent in more vigorous activity (16).

Many device companies have their own, often closed-source, activity endpoints computation, such as ActiGraph,^1^ the Activinsights GENEActiv R markdown scripts,^2^ Axivity,^3^ or the Philips Actiwatch software.^4^ However, using such packages comes with some challenges, notably the lack of generalizability in ingesting data from other devices and the flexibility to incorporate additional configurations. There have been several open-source packages previously released to process accelerometry signals and generate digital metrics, such as GGIR (17) and *pyActigraphy* (18). *pyActigraphy*, with the capability to be used for multiple data formats, but they only provide metrics related to circadian and rest-active rhythms, and not general physical activity or measurements of gait. GGIR, as one of the most commonly used R packages to process and analyze accelerometry signals, has its own limitations. Notably, while GGIR provides many options for its processing steps, understanding these options requires a significant time investment, and any significant modifications to the GGIR’s processing would require intermediate or higher knowledge of R software. Finally, any extensions of GGIR’s functionality would be likely be highly complicated and convoluted due to the current nature of the code base. However, it is still important to keep in mind that GGIR has significant popularity and previous use in literature, and would provide a good benchmark for future physical activity computation packages.

In order to overcome these challenges and limitations, Adamowicz et al. recently released SciKit Digital Health (SKDH) (19), a general-purpose, unified, and open source Python package containing various algorithms (e.g. gait, activity, and sleep), pre-processing steps (e.g. wear detection, signal calibration), and data ingestion methods, that aims to be easily accessible by end users with a minimal learning curve.

In this work, we first present a comparison of SKDH physical activity endpoints against corresponding GGIR physical activity metrics and Activinsights GENEActiv R markdown metrics in a healthy adult cohort. This comparison provides the first hand validation evidence to ensure that the SKDH implementation maintains consistency with existing physical activity implementations that have been widely used to generate physical activity metrics in the literature. Second, we demonstrate the scientific applications of SKDH-generated digital metrics of physical activity by evaluating their relationship with age, with attention to non-threshold based metrics.

## 2. Methods

### 2.1. Subjects and procedure

Data used for validation and testing of the physical activity algorithms were from the Sensors to Record Your Daily Exercise (STRYDE) study completed at the Pfizer Innovation Research Lab (PfIRe Lab) in Cambridge, Massachusetts. Full details of the study design can be found in previous publications (20). In summary, 66 healthy participants were recruited in the greater Boston area, Massachusetts, USA (older cohort (ages 65–85): N=32, 16 Females (50.0%), age 72.3±5.8, younger cohort (ages 23–39) N=33, 17 Females (51.5%), age 29.2±4.6). One male participant in the older adults group was excluded as it was determined that he did not meet the inclusion criteria after study completion. The key inclusion criteria included no significant health issues, BMI between 18.5 and 30 kg/m^2^, or absolute weight <125 kg. The key exclusion criteria included self-reported medical condition, recreational drug use, or medication use preventing study task completion, Vulnerable Elders Survey (21) total score >3 including 0 in all activities of daily living (ADLs)). Participants completed two in-lab visits spaced 7–14 days apart. Between the two lab visits, participants wore accelerometers on their lower back and on their non-dominant wrist while going about their daily lives (all subjects were community-dwelling). Only wrist sensor data are presented in this work. The final protocol and informed consent documentation were reviewed and approved by Advarra Institutional Review Board (study ID: Pro00029419). All participants gave written consent prior to enrollment.

### 2.2. Instrumentation

During the at-home portion of the STRYDE study, participants wore two devices (GENEActiv, Activinsights, UK): one on their non-dominant wrist and another on the lower back. Devices recorded tri-axial accelerometer data (range: ±8 g, sampling rate: 50 Hz) and were attached to the body using straps. Data were stored locally on the device and downloaded for offline processing following the return of the device to the study site. For this work, only data from the wrist worn device were used to generate physical activity metrics.

### 2.3. Reference processing methods

In this work, two different software packages were used as reference benchmarks to process raw accelerometry signals and generate daily physical activity metrics, namely GGIR and the GENEActiv macros.

#### 2.3.1. GENEActiv macros

As the GENEActiv devices were deployed in the study, the accompanying GENEActiv Microsoft Excel Macros (Activinsights now provides these as R markdown scripts) were used as one benchmark processing method to derive physical activity metrics from the at-home monitoring period.

The macros utilize both accelerometry signals and near-body temperature signals and proceed with the following components:

1. Segment the data into 24-hour periods
2. Classify each 24-hour period into periods of non-wear, sleep, sedentary, light, moderate, and vigorous
3. Generate the relevant activity and sleep metrics.
4. Generate reports with tables and visualizations.

#### 2.3.2. GGIR

GGIR was used to compute physical activity metrics due to its wide acceptance and usage in the research community. It has been evaluated in over 90 peer-reviewed journal publications (17). GGIR is a collection of algorithms for activity and sleep research driven by the research community, written in R, and includes code to ingest, calibrate, and detect sleep, and activity levels from raw acceleration data. GGIR’s functionalities consist of the following five groups in logic processing order:

1. Calibrate the raw acceleration, derive the acceleration summary metrics, and detect nonwear time,
2. Generate basic statistical summaries of the acceleration while incorporating the non-wear time,
3. Detect the inactive period based on arm angle variability
4. Detect the sleep period based on the inactive period
5. Derive summaries of sleep and activity metrics and generate the final report.

### 2.4. Proposed method for activity metrics computation in SKDH

There were several key processing steps that happened before the activity metric estimation could occur, notably accelerometer calibration, wear detection, and sleep period detection. While none of these steps are technically necessary to compute activity metrics, they aid in providing more informed values that should better reflect how a person is going about their daily life.

Calibration has been shown to be an important step, especially when computing the activity metrics (22). The goal of the calibration step is to ensure that when a sensor is at rest, it would measure 1 g instead of 1.05 g for example. This was accomplished by mapping all data points reasonably assumed to be at rest to a sphere and then scaling these points to get the least sum of distances from the radius one sphere (22).

The next pre-processing step for the activity metric pipeline was to compute wear time. Using a previously published algorithm (23) called DETACH, acceleration, temperature, and rate of change in temperature were used to determine if a device was being worn. The DETACH algorithm computes wear status with resolutions down to single-digit seconds. The following parameters were used: acceleration standard deviation of 0.008 g, low-temperature threshold of 26.0°C, high-temperature threshold of 30.0°C, temperature rate of decrease threshold of −0.2°C/min, and temperature rate of increase threshold of 0.1°C/min. A window size of 1 s was used, based on the previous work (23). For use cases lacking temperature data, SKDH provides alternative methods of calculating wear time using only the accelerometer data (24–28).

Finally, sleep periods were computed using a modified version of SleepPy (29, 30). This algorithm is very similar to the one used by GGIR (31) and also uses acceleration from the wrist as the basis for estimating sleep periods. In the case where temperature is available (as it is in this work), temperature is also accounted for in determining this sleep window, by excluding periods where the temperature would be uncharacteristically low for worn sleeping periods.

The proposed method for computing physical activity metrics is very similar to that in GGIR and is based on previous work (24, 26, 32) that GGIR uses and cites as well. The algorithm is intuitive and is shown as pseudo-code in Algorithm 1.

**ALGORITHM 1:** Computation of Euclidean Norm Minus One (ENMO) which is then used to compute the rest of the activity metrics.

**Data:** $y_{acc}=[y_{ax},y_{ay},y_{az}]$, day_index, [wear_index], [sleep_index] **Result:** $ENMO_{win}$ **for** day in day_index **do** $y_{acc}\leftarrow y_{acc}[day\_wear]$ // if available **if** $t_{wear}<thresh_{wear}$ **then** continue **end** **for** wake, sleep in day **do** // if available $ENMO(t)\leftarrow \sqrt{y_{ax}^{2}(t)+y_{ay}^{2}(t)+y_{az}^{2}(t)}-1$ ENMO(t)←max(ENMO(t),0) $ENMO_{win}\leftarrow moving\_mean(ENMO,wlen,wlen)$ **end** **end**

In Algorithm 1, $y_{acc}$ is the measured acceleration in units of gravitational acceleration (g), ENMO is the Euclidean Norm Minus One, and $ENMO_{win}$ is the windowed average ENMO over a sub-minute window length designated wlen. For this work, wlen was set to 5 s. Additionally, $thresh_{wear}$ was set to 10 h.

First, iterating over each day (as activity metrics are computed on a per-day basis), the non-wear time was removed. If the remaining acceleration wear time ($t_{wear}$) was less than the $thresh_{wear}$ then no metrics were computed for that day. Next, if sleep periods were provided, the day was split into waking and non-waking (i.e. sleep) periods, and activity metrics were computed separately for each period. Next, ENMO was computed by taking the square root of the sum of the acceleration components squared (Euclidean norm or vector magnitude) and subtracting one for each time point. ENMO was trimmed at 0. Finally, a moving mean with non-overlapping windows was computed to obtain $ENMO_{win}$, which was used as the basis for computing the final activity metrics.

A series of metrics was then produced based on $ENMO_{win}$. The most basic is the accumulation of time in different brackets of activity level. For this work, the following cut-points were used: [0g,0.050g) for sedentary, [0.050g,0.110g) for light, [0.110g,0.440g) for moderate, and [0.440g,∞) for vigorous activity levels (15). From these definitions, moderate and vigorous physical activity (MVPA) was anything above 0.110 g, and sedentary and light physical activity (SLPA) was anything below 0.110 *g*. Additionally, time in bouts (26, 32) were computed for various bout lengths.

While there is much research surrounding the appropriate cut-points to use to segment activity levels (33, 11, 10, 12–15), there has also been some research on moving away from cut-points and time spent in various activity levels. One such method is intensity gradient (16), which instead creates much smaller evenly spaced bins (0.025 g), and summarizes the time in each bin. Taking the natural log of both the mid-point of the bins and the time in each bin allows a linear relationship to be estimated, which gives the intensity gradient (IG), as well as the intercept and r-value for the linear estimation.

Additionally, a series of fragmentation metrics - transition probability, average duration, Gini index, average hazard, and the power law distribution, were computed. While conventional metrics such as sedentary or MVPA times focused on evaluating the total volume of different activity types, these fragmentation metrics, as a general explanation, attempt to evaluate the time accumulation strategy by showing how likely it is for a participant to remain in a particular activity state. Previous work has shown that these metrics were associated with lower mortality risk (34). Definitions for all metrics are included in Activity Endpoint Definitions (Appendix 1).

All processing for this method was performed using Scikit Digital Health (19), a Python package for wearable inertial sensor data processing, version 0.11.2.

### 2.5. Statistical methods

Physical activity metrics generated by SKDH were compared to reference metrics generated by GGIR and GENEActiv macros. Specifically, the analyses focus on metrics that are shared across the platforms. For such comparisons, both mean metrics across days and the repeated days of measurements were considered. To compare the mean across days, pairwise t-tests, intra-class correlation coefficients (ICC), and Pearson correlation coefficients were calculated. Furthermore, to incorporate day-to-day variability in daily physical activity, repeated measures correlation was also computed, which accounts for non-independence among observations using analysis of covariance to statistically adjust for inter-individual variability (35, 36). The difference between each pair across repeated days was fitted into a mixed effects model as response and subject as the random effect. The estimated mean differences were reported for each pair.

The relationship between age and SKDH-generated physical activity metrics was explored from two aspects. Activity metrics were averaged across days for each participant. First, to investigate the overall between-group difference in each of the activity metrics, two group t-tests were performed. The standardized effect size (Cohen’s d) was computed to quantify the difference between the two age groups. As a convention, Cohen’s d can be used to classify effect sizes as small (d=0.2), medium (d=0.5), and large (d≥0.8) (37).

In gerontology, trajectories of measurements of interests are typically fitted as a function of age to study the measurements across the lifespan (38). Therefore, for each activity metric, we fitted two separate linear regressions with age as the fixed effect, one for each age group. The estimated age effects (i.e. the estimated slope coefficient for age) between the two groups were then compared based on calculated t-statistics.

All statistical analysis was performed in R version 4.2.1 with the following packages: “nlme” for mixed model with repeated measures (MMRM), “rmcorr” for repeated measure correlation, and “psych” for ICC. The group analysis p-values were corrected for multiple comparisons using false discovery rate correction.

## 3. Results

### 3.1. Comparison between SKDH and GGIR/GENEActiv macros

We assessed the reliability of activity metrics derived using SKDH algorithm, by comparing them with activity metrics provided by GGIR and GeneActiv macros. Table 1 displays the comparison of averaged activity metrics across days derived from SKDH, with respect to the GGIR and GeneActiv Macro through ICC, mean difference and correlation. The ICC and correlation between SKDH and GGIR showed great agreement for most activity metrics (ICC>0.75), except for sedentary time, possibly due to slightly different definitions of sleep in these two algorithms. On the contrary, metrics generated from GeneActiv macros algorithm were classified based on different threshold definitions for varying activities, thus presented high correlation with SKDH algorithm but with large discrepancy in ICC values.

**Table 1:** Comparisons of mean activity metrics across days between SKDH and references (GGIR/GENEActiv Macros) for selected activity metrics.

Package	Metrics	ICC (95% CI)	Mean diff. (p-value*)	Corr. (p-value)
GGIR	Intensity gradient	0.941 (0.048,0.986)	−0.083 (<0.001)	0.989 (<0.001)
	MVPA time	0.997 (0.991,0.999)	1.900 (<0.001)	0.998 (<0.001)
	Sedentary time	0.550 (0.356,0.699)	−2.299 (0.8162)	0.547 (<0.001)
	Light time	0.995 (0.991,0.997)	0.968 (0.0315)	0.995 (<0.001)
	Moderate time	0.997 (0.996,0.998)	0.748 (0.0472)	0.998 (<0.001)
	Vigorous time	1.000 (1.000,1.000)	−0.001 (0.9393)	1.000 (<0.001)
GENEActiv macros	Sedentary time	0.530 (−0.056, 0.793)	−76.517 (<0.001)	0.733 (<0.001)
	Light time	0.469 (−0.032, 0.813)	−45.040 (<0.001)	0.957 (<0.001)
	Moderate time	0.618 (−0.067, 0.880)	48.954 (<0.001)	0.947 (<0.001)
	Vigorous time	0.697 (0.452,0.828)	3.233 (<0.001)	0.812 (<0.001)
	Max. acc. 15 min	—^†^	—^†^	0.967 (<0.001)

* *p*−values were computed from pairedt−tests.

^†^ Incompatible units: the acceleration summaries are different in units therefore the ICC and mean difference are not appropriate to be calculated.

*Max. acc.*,maximum acceleration.

The results of comparisons based on repeated measurements were presented in the Supplemental Data, which are consistent with the results Table 1 based average metrics.

### 3.2. The relationship between age and SKDH-generated activity metrics

Table 2 shows relationship between selected physical activity metrics and age, ordered by the absolute values Cohen’s d, which measures the effect size of the difference between two means. The results for all derived metrics can be found in Supplemental Data. Statistically significant differences between younger and older age groups were observed for multiple physical activity metrics. In particular, large effect sizes (Cohen’s d>0.8) were observed for SLPA transition probability, intensity gradient, MVPA, moderate activity time, IG intercept, and maximum 15-min window accelerations.

**Table 2:** The association between age and selected SKDH-derived physical activity metrics.

	Group mean (SD)				
	Younger	Older	|Cohen’s d|	p-value (mean)	p-value (slope)
SLPA trans. prob.	0.04 (0.01)	0.02 (0.01)	1.68	<0.001	0.409
Intensity gradient	−2.32 (0.18)	−2.58 (0.23)	1.27	<0.001	0.017
MVPA time [min]	98.37 (36.56)	57.63 (30.88)	1.22	<0.001	0.130
Moderate time [min]	93.08 (35.76)	55.58 (28.96)	1.17	<0.001	0.167
Max. acceleration 6 min [g]	0.32 (0.14)	0.20 (0.09)	1.04	<0.001	0.185
IG intercept	13.48 (0.79)	14.25 (0.76)	1.00	<0.001	0.153
Max. acceleration 15 min [g]	0.24 (0.11)	0.16 (0.08)	0.87	0.001	0.076
Vigorous time [min]	5.29 (7.61)	2.05 (3.26)	0.56	0.030	0.332
MVPA trans. prob.	0.43 (0.11)	0.51 (0.20)	0.54	0.038	<0.001
Sedentary time [min]	708.40 (82.29)	732.94 (86.79)	0.29	0.247	0.095
Light time [min]	118.27 (25.36)	118.89 (41.09)	0.02	0.942	0.180

**Figure 1:**
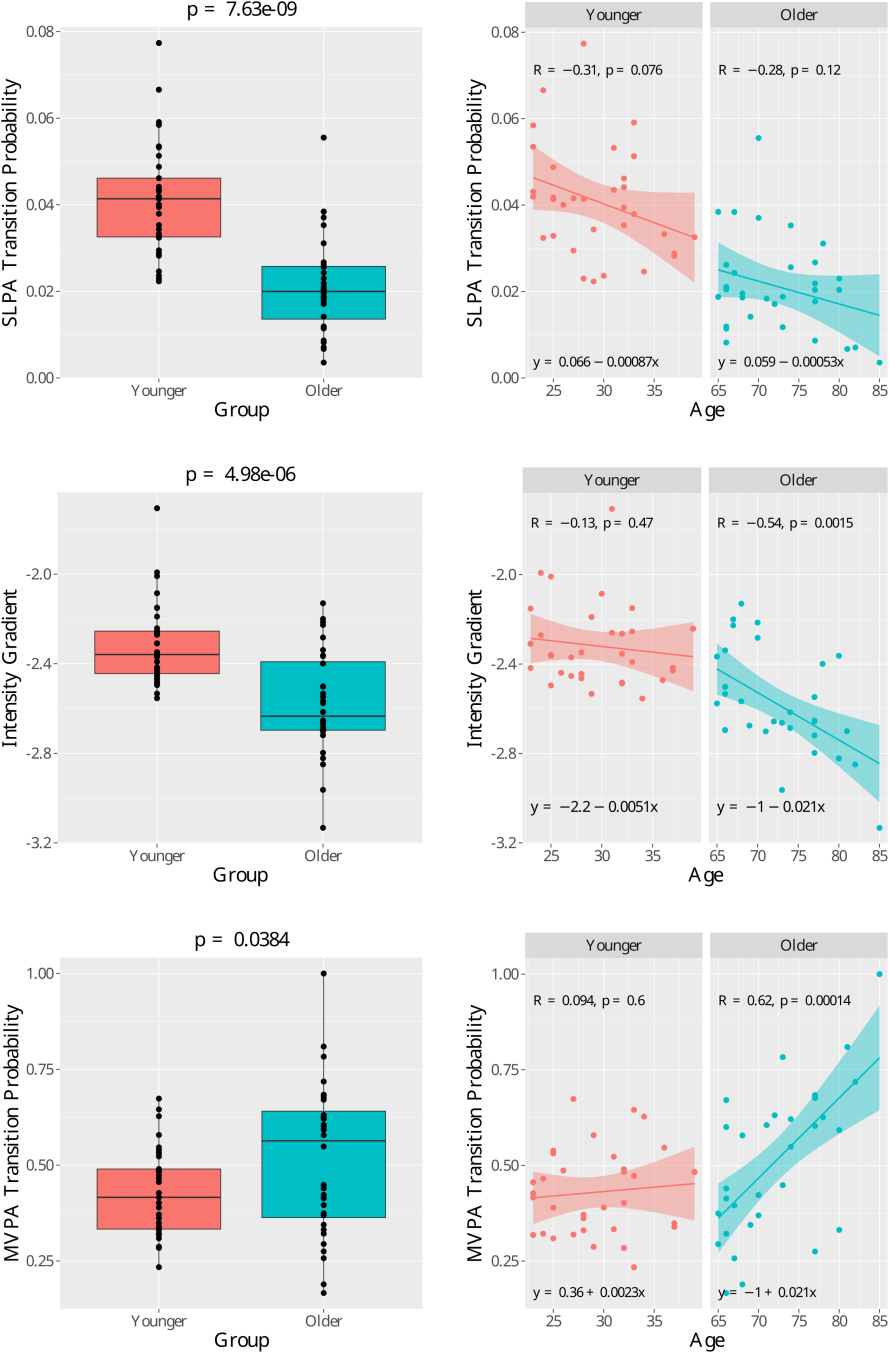
Age group comparison for three features with high Cohen’s d effect size. (Top left) Boxplot of the sedendary/light to moderate/vigorous physical activity (SLPA) transition probability. Group mean t-test *p*-value = 7.36×10^−9^. (Top right) Scatter plots for SLPA transition probability for each age group with regression lines. (Mid left) Boxplot of the intensity gradient. Group mean *t*-test *p*-value = 4.98×10^−6^.(Mid right) Scatter plots for intensity gradient for each age group with regression lines.(Bottom left) Boxplot of the moderate∕vigorous to sedendary∕light physical activity (MVPA) transition probability. Group mean t− test p-value=0.0384.(Bottom right)Scatter plots for MVPA transition probability for each age group with regression lines. In the right graphs, R is the Pearson correlation coefficient and pis thep− value for regression slope coefficient.

Group mean (SD): the group means and the corresponding standard deviations; *Cohen’s d*: a measure of effect size of the difference. Cohen classified effect sizes as small (d=0.2), medium (d=0.5), and large (d≥0.8); *p value (mean)*: the p values for the two group t-tests to compare the group mean difference; *p-value (slope)*: the p values for the comparison of the age effects (slope coefficient of age in regression models) between the two age groups (see Figure 1); *SLPA*: Sedentary & light physical activity; *MVPA*: moderate & vigorous physical activity; *SLPA trans. prob.*: Transition probability out of sedentary/light to moderate/vigorous states; *MVPA trans. prob.*: transition probability out of moderate/vigorous to sedentary/light states.

Notably, the fragmentation metrics SLPA transition probability (interpreted as the transition probability out of sedentary/light states to moderate/vigorous states) shows the largest between-group difference. Time spent in moderate activity and MVPA showed strong evidence of a group difference (Cohen’s d>1.17), while vigorous time alone along with sedentary and light activity showed no significant differences between younger and older age groups (Cohen’s d<0.56).

Regarding the difference in the age effects (i.e. slope estimation) between the two groups, among the metrics listed in Table 2, it was observed that the age effects for intensity gradient (p-value 0.017) and MVPA transition probability (p-value<0.001) are different. The effects can be observed in Figure 1. A clear “hockey stick” shape can be observed where the younger age shows a stable pattern but the older age shows an accelerated change. In contrast, even though there is significant difference in SLPA transition probability between the two age groups, the age slopes between the two groups show difference, and thus we observe a rather “linear” decline instead of a “hockey stick” shape. Among all the metrics generated by SKDH (Supplemental Data), additional metrics showing evidence of age slope differences between groups include MVPA accumulated in 10min bout time (p-value 0.038), SLPA average duration (p-value 0.001), and SLPA average hazard and power law distribution.

## 4. Discussion

We introduced the open-source Python package, Scikit Digital Health (SKDH) (19), and how it can be used to generate physical activity metrics. We provided a comparison between the SKDH-generated metrics against existing methods, i.e. GGIR and GENEActiv Macros using data collected from a study in healthy adults. We further examined the association between the generated activity metrics with age, which is crucially related to epidemiological and health-related research (39).

When comparing SKDH-generated metrics with the references, correlations were generally strong. For both reference algorithms, relatively larger bias and lower correlation were observed for sedentary activity time. One possible explanation for this discrepancy is due to the use of slightly different sleep algorithms, which potentially cause over- or under-estimation of sedentary time since they share similar patterns, impacting these comparisons. Specifically, there is excellent agreement between SKDH and GGIR. This is primarily because that the SKDH and GGIR shared the same list of algorithms to generate the metrics, namely using ENMO as an acceleration aggregation metric, and the use of the same thresholds for the defined activity levels. Between SKDH and the GENEActiv Macros, we observed moderate ICC values and larger biases, which presumably is driven by a different acceleration accumulation algorithm, and different thresholds for the sedentary, light, moderate, and vigorous activity levels used. It is worth mentioning that, neither of GGIR and GENEActiv macros should be considered as “ground truth”. Therefore, our comparison is technically not a “analytical validation” according to the V3 framework (40). However, what is shown in this paper is still extremely valuable as they demonstrate that SKDH’s results agree with other widely accepted tools. It deserves to be highlighted that in this work, as opposed to most of other validation works which typically average the repeated measurements across days, we incorporated the day-to-day variation using repeated measure regression models. Even though the results are consistent in this population who are healthy, it is still highly recommended to consider the repeated measure design for future analyses.

Significant differences between the younger and older age groups were found for several metrics including sedentary to active transition probability, intensity gradient, moderate and vigorous physical activity time, and maximum 6-min acceleration, which all showed Cohen’s d over 1.0. Since age is a key risk factor for a wide variety of diseases and health conditions (39), there is potential to deploy these digitally measured physical activity metrics as novel digital endpoints in clinical trials as they may be able to provide large effect sizes with smaller sample sizes than typical for these clinical trials. While metrics such as MVPA, sedentary, and light physical activity time are threshold based, with thresholds changing from study to study (10–15), and changing between children and adults (14, 11, 10), the intensity gradient (16) method does not rely on these thresholds. The observed between-group differences for intensity gradient, therefore, provides evidence that non-threshold activity metrics can be utilized in future studies with fewer challenges to interpreting results based on a common set of thresholds for potentially diverse participant groups. Future work could focus on exploring additional non-threshold-based methods for calculating activity endpoints.

Even though there are significant between group differences for metrics such as the transition probability from sedentary/light to moderate/vigorous states, over the life span, those metrics are monotonically increasing or declining. On the contrary, we identified the difference in the age effects (i.e. slope estimation) between the two age groups for a variety of activity metrics such as intensity gradient and MVPA transition probability. We observed a “hockey stick” shape over a lifespan—the trajectories tend to be stable and flat in younger ages and suddenly come with steeper and accelerated change in older ages. Take MVPA transition probability, which indicates the likelihood to transition from MVPA to light activity or sedentary behaviors (i.e. to break up high-intensity activity), as an example. We observed that, between the ages of 23 to 39, participants’ likelihood to break up high-intensity activity tends to stay stable, while between the ages of 65 to 85, the likelihood drastically increases, which indicates a serious decline in the ability to sustain high-intensity activity. The “hockey stick” trajectories we identified are consistent with previous publication on the age-caused decline in mobility and cognitive function and is a key concept in gerontology to study the biology of aging and the determination of biomarkers to track these effects (41–43). Notably, the difference in the age effects (i.e. slope estimation) between the two age groups was majorly identified for metrics that quantify activity fragmentation, which is a relatively new concept since most research is still focused on the use of metrics to quantify total activity volumes such as MVPA and total sedentary time. Such findings are consistent with growing literature demonstrating the value of using fragmentation metrics that explore the pattern to accumulate active and sedentary time. These metrics have been shown to be associated with functional measurements of mobility, all-cause mortality, and hearing loss (44, 34, 45–49).

There are a few limitations with this work, namely that the sample size was relatively small compared to large cohort studies that quantify physical activity measurements. It is aligned with our goal to eventually apply SKDH to large cohort studies in the future. Additionally, even slightly different sleep algorithms potentially resulted in a biased estimation of sedentary and light PA due to the misclassification of sedentary activity. Future work may be needed to increase the specificity of the sleep detection algorithm. The analyses were conducted only in healthy adults, and future work should focus on incorporating pathological participants who may have different patterns of physical activity. While this work focused on the wrist location, which is commonly used for physical activity estimation due to ease and comfort of wearing (50), exploring the lumbar sensor available in this study is a potential avenue for future work as well. Finally, while we present two different reference methods, there are other commonly used algorithms to assess physical activity, such as algorithms based on ActiGraph’s activity counts, the algorithms for Axivity, and the Philips Actiwatch. Actigraph’s recent move to publicize its proprietary activity counts algorithm (51) increases the possibility of applying their algorithms and analytic platforms for other devices, which remains an avenue of exploration for future work.

## Data availability statement

The original contributions presented in the study are included in the article/Supplementary Materials, further inquiries can be directed to the corresponding author.

## Ethics statement

The studies involving humans were approved by Advarra Institutional Review Board. The studies were conducted in accordance with the local legislation and institutional requirements. The participants provided their written informed consent to participate in this study.

## Footnotes

^1^ https://theactigraph.com/centrepoint

^2^ https://activinsights.com/support/geneactiv-support/

^3^ https://axivity.com/

^4^ http://www.actigraphy.respironics.com/solutions/actigraphy.aspx

## Appendix 1. Activity endpoint definitions

**Table:**

Intensity gradient	A measure of the decrease in time spent as intensity of activity increases. Calculated by taking the log of time spent in 25 mg bins and finding the slope of these points (16).
Maximum acceleration	A measure of the maximum mean acceleration in a window of time. Calculated by taking the mean acceleration in windows through a day, and taking the highest mean acceleration for that day.
Bout time	A modified accounting of time spent at a particular activity intensity level, where only time spent in bouts of a minimum length (typically 1, 5, or 10 min) is counted.
Average duration	The average duration spent in a particular intensity of activity. Calculated by obtaining the bout lengths of an activity intensity and taking the mean of these bout lengths (34).
Transition probability	A measure of the likelihood to transition out of a particular activity intensity. The computation for this, through some manipulation ends up being the inverse of the Average Duration (34).
Gini index	A measure of the variability of the bout lengths. Normalized from 0 to 1, such that values near 1 indicate that the total time was accumulated mostly due to a small number of large bouts, while values near 0 indicate that the total time was accumulated through a large number of relatively similar length bouts (34).
Average hazard	A measure summarizing the frequency of transitioning from one state to another. Higher values indicate higher frequency in switching between states (34). (A1) $$h(t_{n_{i}})=\frac{n(t_{n_{i}})}{n-n^{c}(t_{n_{i-1}})}$$ (A2) $$\hat{h}=\frac{1}{m}\sum_{t\in D}h(t)$$ where $h(t_{n_{i}})$ is the hazard for the bout of length $t_{n_{i}}$, $n(t_{n_{i}})$ is the number of bouts of length $t_{n_{i}}$, n is the total number of bouts, $n^{c}(t_{n_{i}})$ is the sum bumber of bouts less than or equal to length $t_{n_{i}}$, and t∈D indicates all bouts up to maximum length D.
Power law distribution	The α from the power law distribution. Larger values indicate that the total time is accumulated with a larger portion of short length bouts, i.e. more frequent transitions. Computed by (A3) $$\alpha =1+\frac{n}{\sum_{i}\log t_{i}/(\min(t)-0.5)}$$ where n is the number of bouts, $t_{i}$ is the duration of the ith bout, and min(t) is the length of the shortest bout (34).
