# Supplementary material for: SciKit digital health package for accelerometry-measured physical activity: comparisons to existing solutions and investigations of age effects in healthy adults
Source: Front Digit Health. 2023 Nov 27;5:1321086. doi: 10.3389/fdgth.2023.1321086 (PMC10715430; doi:10.3389/fdgth.2023.1321086)
Supplement: Supplementary file 1 [file Datasheet1.pdf]

Supplementary Material

1 COMPARISON BETWEEN SKDH AND GGIR/GENEACTIV MACRO USING MULTI-LEVEL DATA

Table S1. Comparisons of daily activity metrics between SKDH and references (GGIR / GENEActiv Macros) for selected activity metrics using multi-level methods.

Package	Metrics	Rep Corr. * (p-value)	Mean Diff. **(p-value)
GGIR	Intensity Gradient	0.951 (<0.001)	-0.082 (<0.001)
	MVPA Time	0.995 (<0.001)	1.917 (<0.001)
	Sed. Time	0.568 (<0.001)	-3.264 (0.735)
	Light Time	0.984 (<0.001)	0.975 (0.027)
	Mod. Time	0.995 (<0.001)	0.751 (0.040)
	Vig. Time	1.000 (<0.001)	0.000 (0.994)
GENEActiv Macros	Sed. Time	0.629 (<0.001)	-76.028 (<0.001)
	Light Time	0.863 (<0.001)	-45.423 (<0.001)
	Mod. Time	0.912 (<0.001)	49.567 (<0.001)
	Vig. Time	0.846 (<0.001)	3.254 (<0.001)
	Max. Acc. 15min	0.964 (<0.001)	-†

† Incompatible units: the acceleration summaries are different in units therefore the mean difference are not appropriate to be calculated;
Sed: Sedentary *Mod*: Moderate *Vig*: Vigorous
Max. Acc: Maximum acceleration * Repeated measure correlation
** Mean differences estimated based on mixed effects model

2 FULL AGE COMPARISON RESULTS

Table S2: Comparison between younger and older healthy adult cohorts using selected activity features, ordered by absolute Cohen's d. Absolute Cohen's d is a measure of the difference in group means. Additionally the p-value (mean) is also assessing the group means, while the p-value (slope) is assessing the difference in trend-lines between the age groups.

	Group Mean (SD)				
	**Younger**	**Older**	**$\lvert$Cohen's $d\rvert$**	**p-value (mean)**	**p-value (slope)**
IG	-2.32 (0.18)	-2.58 (0.23)	1.27	< 0.001	0.017
IG Intercept	13.48 (0.79)	14.25 (0.76)	1.00	< 0.001	0.153
IG r^2	0.87 (0.04)	0.88 (0.03)	0.28	0.2639	0.78
Max. Acc. 6min [g]	0.32 (0.14)	0.20 (0.09)	1.04	< 0.001	0.185
Max. Acc. 15min [g]	0.24 (0.11)	0.16 (0.08)	0.87	0.001	0.076
Max. Acc. 60min [g]	0.14 (0.07)	0.10 (0.05)	0.71	0.006	0.166
MVPA Time [min]	98.37 (36.56)	57.63 (30.88)	1.22	< 0.001	0.130
Sed. Time [min]	708.40 (82.29)	732.94 (86.79)	0.29	0.247	0.095
Light Time [min]	118.27 (25.36)	118.89 (41.09)	0.02	0.942	0.180
Mod. Time [min]	93.08 (35.76)	55.58 (28.96)	1.17	< 0.001	0.167
Vig. Time [min]	5.29 (7.61)	2.05 (3.26)	0.56	0.030	0.332
MVPA 1min Bout Time [min]	54.66 (34.50)	29.47 (23.35)	0.87	0.001	0.133
MVPA 5min Bout Time [min]	39.33 (34.16)	22.29 (21.70)	0.60	0.019	0.059
MVPA 10min Bout Time [min]	31.80 (33.77)	17.50 (19.69)	0.52	0.041	0.038
Sed. 1min Bout Time [min]	676.26 (87.59)	703.21 (96.00)	0.30	0.242	0.169

Continued on next page

Table S2 – continued from previous page

	Group Mean (SD)				
	**Younger**	**Older**	**|Cohen's d|**	**p-value (mean)**	**p-value (slope)**
Sed. 5min Bout Time [min]	668.17 (94.65)	700.08 (103.92)	0.33	0.201	0.222
Sed. 10min Bout Time [min]	664.01 (100.06)	702.78 (107.50)	0.38	0.138	0.243
Light 1min Bout Time [min]	15.74 (7.24)	24.95 (14.15)	0.84	0.002	0.594
Light 5min Bout Time [min]	1.43 (2.28)	5.25 (7.74)	0.68	0.011	0.179
Light 10min Bout Time [min]	0.57 (1.23)	3.18 (6.44)	0.58	0.031	0.681
Mod. 1min Bout Time [min]	47.86 (34.27)	26.47 (20.98)	0.76	0.004	0.166
Mod. 5min Bout Time [min]	32.65 (33.78)	18.99 (19.38)	0.50	0.050	0.077
Mod. 10min Bout Time [min]	26.37 (33.26)	14.72 (17.54)	0.44	0.082	0.065
Vig. 1min Bout Time [min]	3.22 (6.29)	0.62 (1.77)	0.57	0.028	0.401
Vig. 5min Bout Time [min]	2.39 (5.08)	0.20 (0.79)	0.61	0.020	0.499
Vig. 10min Bout Time [min]	1.61 (3.61)	0.09 (0.49)	0.59	0.022	0.452
Sed. Avg. Dur. [min]	11.88 (3.25)	16.31 (7.22)	0.81	0.003	0.704
Sed. Trans. Prop.	0.10 (0.02)	0.08 (0.03)	0.64	0.014	0.604
Sed. Gini Index	0.66 (0.02)	0.68 (0.03)	1.01	< 0.001	0.307
Sed. Avg. Hazard	0.21 (0.02)	0.22 (0.02)	0.43	0.089	0.296
Sed. Power Law Distribution	1.46 (0.04)	1.44 (0.05)	0.46	0.075	0.518
SLPA Avg. Dur. [min]	29.72 (10.60)	83.77 (58.24)	1.32	< 0.001	0.001
SLPA Trans. Prob.	0.04 (0.01)	0.02 (0.01)	1.68	< 0.001	0.409
SLPA Gini Index	0.68 (0.04)	0.70 (0.06)	0.31	0.221	0.055
SLPA Avg. Hazard	0.21 (0.02)	0.31 (0.10)	1.43	< 0.001	0.002

Continued on next page

Table S2 – continued from previous page

	Group Mean (SD)				
	**Younger**	**Older**	**|Cohen's d|**	**p-value (mean)**	**p-value (slope)**
SLPA Power Law Distribution	1.36 (0.03)	1.43 (0.23)	0.45	0.087	< 0.001
MVPA Avg. Dur. [min]	2.77 (0.98)	2.85 (1.59)	0.06	0.816	0.001
MVPA Trans. Prob.	0.43 (0.11)	0.51 (0.20)	0.54	0.038	< 0.001
MVPA Gini Index	0.44 (0.08)	0.42 (0.16)	0.18	0.48	0.631
MVPA Avg. Hazard	0.55 (0.09)	0.66 (0.14)	0.96	< 0.001	0.005
MVPA Power Law Distribution	1.82 (0.11)	1.92 (0.20)	0.61	0.020	0.007
Sleep MVPA Time [min]	9.90 (17.84)	1.93 (4.49)	0.62	0.018	0.611
Sleep Sed. Time [min]	360.69 (136.20)	254.36 (161.20)	0.72	0.006	0.354
Sleep Light Time [min]	12.92 (19.70)	6.42 (17.61)	0.35	0.166	0.710
Sleep Mod. Time [min]	9.01 (15.68)	1.92 (4.43)	0.62	0.017	0.442
Sleep Vig. Time [min]	0.90 (3.91)	0.01 (0.06)	0.32	0.203	0.428

Trans. Prob.: Transition probability
Sed: Sedentary
Mod: Moderate
Vig: Vigorous
SLPA: Sedentary & light physical activity
MVPA: Moderate & vigorous physical activity
Max. Acc: Maximum acceleration
Avg. Dur: Average duration
IG: Intensity gradient
